# Serum Galectin-3 Predicts Mortality in Venoarterial Extracorporeal Membrane Oxygenation Patients

**DOI:** 10.1155/2023/3917156

**Published:** 2023-09-30

**Authors:** Jianlong Zhu, Dehui Guo, Liying Liu, Jing Zhong

**Affiliations:** ^1^Department of Critical Care Medicine, Ganzhou People's Hospital, Ganzhou 341000, China; ^2^Department of Critical Care Medicine, Quannan People's Hospital, Ganzhou 341000, China; ^3^Department of Blood Transfusion, Ganzhou People's Hospital, Ganzhou 341000, China

## Abstract

**Objective:**

We investigated the potential use of galectin-3 (Gal-3) as a prognostic indicator for patients with cardiogenic shock and developed a predictive mortality model for venoarterial extracorporeal membrane oxygenation (VA-ECMO).

**Methods:**

We prospectively studied patients (survivors and nonsurvivors) who received VA-ECMO for cardiogenic shock from 2019 to 2021. We recorded baseline data, Gal-3, and B-type natriuretic peptide (BNP) before ECMO and 24–72 h after ECMO. We used multivariable logistic regression to analyze significant risk factors and construct a VA-ECMO death prediction model. Receiver operating characteristic (ROC) curves were plotted to assess the predictive efficacy of the model.

**Results:**

We enrolled 73 patients with cardiogenic shock who received VA-ECMO support; 38 (52.05%) died in hospital. The median age was 57 years (interquartile range (IQR): 48–67 years); the median duration of ECMO therapy was 5.8 days (IQR: 4.62–7.57 days); and the median intensive care unit stay was 19.04 days (IQR: 13.92–26.15 days). Compared with the nonsurvivors, survivors had lower acute physiology and chronic health evaluation (APACHE) II scores (*p* < 0.001), increased left ventricular ejection fraction (*p* < 0.05), lower Gal-3 levels at 24 and 72 h (both *p* = 0.001), lower BNP levels at 24 and 72 h (both *p* = 0.001), and higher platelet counts (*p* = 0.009). Further multivariable analysis showed that APACHE II score, BNP-T72, and Gal-3-T72 were independent risk factors for death in VA-ECMO patients. Gal-3 and BNP were positively correlated (*p* < 0.05) and decreased significantly during ECMO treatment. The areas under the ROC curve (AUC) for APACHE II score, Gal-3-T72, and BNP-T72 were 0.687, 0.799, and 0.723, respectively. We constructed a combined prediction model with an AUC of 0.884 (*p* < 0.01).

**Conclusion:**

Gal-3 may serve as a prognostic indicator for patients receiving VA-ECMO for cardiogenic shock. The combined early warning score is a simple and effective tool for predicting mortality in VA-ECMO patients.

## 1. Introduction

Cardiogenic shock (CS) is a complex clinical syndrome characterized by impaired cardiac function and end-organ hypoperfusion [1, 2]. Studies have shown that cardiogenic shock develops in 5–13% of patients with acute myocardial infarction (AMI) [3]. CS can be caused by various factors, such as AMI, myocarditis, cardiomyopathy, pericardial tamponade, or postcardiac surgery. In Europe, approximately 60,000–70,000 patients experience AMI each year. Despite significant advances in interventional treatment and critical care, mortality from infarct-related CS remains high, reaching 50% within the first 30 days of hospitalization [4]. Extracorporeal membrane oxygenation (ECMO) has become the primary rescue therapy for patients with severe CS. By providing adequate blood flow and oxygen supply through venoarterial extracorporeal membrane oxygenation (VA-ECMO), the damaged heart can rest and recover, leading to improved symptoms and prognosis in patients with CS.

Galectin-3 (Gal-3) is a multifunctional protein that plays a crucial role in many physiological and pathological processes, such as cell growth, differentiation, apoptosis, cell adhesion, angiogenesis, inflammation, fibrogenesis, and tumor progression. Recent studies have shown that Gal-3 is the most overexpressed protein in heart failure (HF) [5]. It can predict the progression of HF and guide its treatment. Sharma et al. found that Gal-3 is the most differentially regulated gene associated with HF [6]. Gal-3 induces the proliferation of fibroblasts and causes heterogeneous deposition of different types of collagen, ultimately leading to loss of cardiac function. Therefore, Gal-3 may be a novel biomarker for predicting circulatory failure.

Cardiovascular disease is a specialty of our hospital, and more than half of our ECMO patients suffer from this condition. Therefore, developing a predictive model for patients with CS is crucial. The prognostic value of Gal-3 for patients with CS undergoing VA-ECMO has not yet been established. In this study, we aimed to investigate the association between Gal-3 levels and the clinical outcomes in patients receiving VA-ECMO.

## 2. Methods

This study was a prospective trial and all procedures followed ethical guidelines. All study subjects or their family members gave informed consent for the treatment protocol. The study enrolled patients with cardiogenic shock who received VA-ECMO at Ganzhou People's Hospital from January 2019 to December 2021. All included subjects had class IV cardiac function according to the New York Heart Association (NYHA) classification. Our study estimated the Gal-3 levels before ECMO treatment and at 72 h for surviving and deceased patients. Based on the expected mean difference, standard deviation, and quantile values, we calculated that a total sample size of 58 was needed for our randomized controlled trial. To improve the reliability and stability of the study results, our team enrolled 73 patients. Patients were categorized into a survivor group and a nonsurvivor group based on their outcome during hospitalization.

The inclusion criteria for this study were as follows: for patients who met the diagnostic criteria for CS and received VA-ECMO at our institution: (1) patients aged over 10 years, as our hospital currently lacks a pediatric ECMO circuit and could not provide ECMO support for younger patients; (2) presence of severe underlying heart disease (such as extensive myocardial infarction, myocarditis, arrhythmia, mechanical valve failure, or postcardiac surgery); (3) presented with typical clinical signs of shock, including persistent hypotension, oliguria, altered consciousness, and other symptoms; (4) after active fluid resuscitation, hypotension and clinical signs did not improve or worsened; or (5) hemodynamic parameters meet the following typical characteristics: (i) systolic blood pressure (SBP) ≤ 90 mmHg or mean arterial pressure drop ≥30 mmHg, or a decrease of 60 mmHg from the baseline systolic blood pressure in hypertension patients for at least 30 min; (ii) central venous pressure is normal or high; (iii) elevated left ventricular end-diastolic pressure or pulmonary capillary wedge pressure; and (iv) reduced cardiac output, and a cardiac index (CI) ≤ 2.2 [L/min]/m^2^; and (6) informed consent from the patient or their family members for VA-ECMO treatment.

The exclusion criteria were as follows: (1) age <10 years or > 85 years; (2) chronic kidney disease, cirrhosis, or a history of malignancy; (3) infectious diseases unrelated to the heart; (4) death within 3 days; (5) voluntary withdrawal or incomplete information; and (6) VA-ECMO conversion to venoarterial-venous (VAV)-ECMO or venovenous (VV)-ECMO.

### 2.1. Data Collection

Basic information including gender, age, underlying disease, cause of cardiogenic shock, body mass index (BMI), and the acute physiology and chronic health evaluation (APACHE) II score were recorded for all patients before VA-ECMO. Venous blood samples were collected at three time points (before ECMO initiation, 24 h after ECMO initiation, and 72 h after ECMO initiation) to measure Gal-3, B-type natriuretic peptide (BNP), platelet, and lactate levels. The durations of ECMO support and intensive care unit (ICU) stay were recorded, and patients were followed up by telephone 28 days after ECMO discontinuation.

### 2.2. VA-ECMO Therapy

Before ECMO initiation, we assessed the patient's vascular status by ultrasound to select an appropriate puncture site and catheter size. An arterial and a venous cannula were inserted under ultrasound guidance by a specialist, and the cannula positions were verified by ultrasound or X-ray. We used a standard oxygenator (MAQUET, Germany) that was primed with saline and connected in a sterile manner after successful cannulation of the artery and the vein. ECMO blood flow and oxygen sweep gas flow were adjusted according to the mean arterial pressure and peripheral oxygen saturation to maintain a mean arterial pressure above 65 mmHg. For systemic anticoagulation, the initial bolus dose of heparin sodium was 50 U/kg, and the maintenance infusion dose was 5–15 [U/kg]/h. The target range of the activated partial thromboplastin time (APTT) was 55–65 s.

### 2.3. Statistical Method

Statistical analysis was performed using SPSS 25.0 software. Continuous variables with normal distribution were expressed as mean ± standard deviation (mean ± SD) and were compared by the independent-samples *t*-test between groups. Continuous variables with non-normal distribution were expressed as median (interquartile range) and were compared by the Mann–Whitney *U*-test between groups. Categorical variables were tested using the chi-square test or Fisher's exact test, as appropriate. Backward stepwise logistic regression was used for mortality risk factor analysis and predictive modeling. The receiver operating characteristic (ROC) curve was used to determine the optimal cut-off values of the risk factors, and the accuracy of each risk factor and prediction model in predicting mortality in ECMO patients was evaluated by calculating the area under the curve (AUC). A *p* value <0.05 was considered statistically significant.

## 3. Results

### 3.1. Demographics and Characteristics

**Figure 1:**
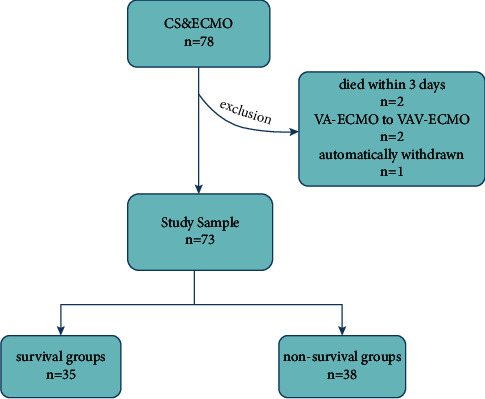
Flow diagram for screening patients who met the inclusion criteria.

A total of 78 patients with CS undergoing VA-ECMO were enrolled in this study, with one automatic dropout, two deaths within 3 days, and two cases transferred from VA-ECMO to VAV-ECMO treatment. Therefore, the final analysis included 73 cases (shown in Figure 1), with 35 (47.95%) in the survivor group and 38 (52.05%) in the nonsurvivor group. The median age of the patients was 57 (48–67) years, the median ECMO treatment time was 5.8 (4.62–7.57) days, and the median ICU stay was 19.04 (13.92–26.15) days. The top three disease categories for performing VA-ECMO were AMI (32.88%), heart valve disease (28.77%), and aortic coarctation type A (21.92%). Among them, 33 (45.21%) cases were after open-heart surgery and 17 (23.29%) were percutaneous coronary intervention patients (as shown in Table 1).

There was no statistically significant difference in age and sex between the survivor and nonsurvivor groups of ECMO patients. There was no difference in Gal-3 and BNP levels between the two groups before ECMO initiation. However, after ECMO treatment, Gal-3 and BNP levels at 24 and 72 h were lower in the survivor group than in the nonsurvivor group, which was statistically significant (*p* < 0.05). Platelet counts were below normal in both groups but were higher in the survivor group than in the nonsurvivor group, which was statistically significant (*p* < 0.05). There was no statistical significance between the two groups in terms of lactate levels at 72 h after VA-ECMO initiation, duration of ECMO support, and length of ICU stay (*p* > 0.05) (as shown in Table 1).

## 4. Effects of VA-ECMO on BNP and Gal-3

The Friedman test for repeated measures was used to compare Gal-3 and BNP levels at three time points in the study subjects. There were significant differences among Gal-3 levels at baseline, 24 h, and 72 h (*χ*^2^ = 134.33, *p* < 0.01), with mean ranks of 2.95, 2.03, and 1.03, respectively. There were also significant differences among BNP levels at baseline, 24 h, and 72 h (*p* < 0.01), with mean ranks of 3.00, 1.52, and 1.48, respectively. Post hoc analysis with the Wilcoxon signed-rank test showed that BNP levels decreased significantly from baseline to 24 h and from baseline to 72 h, but not from 24 h to 72 h (*p* > 0.05), as shown in Table 2.

## 5. The Relationship between Gal-3 and BNP

**Figure 2:**
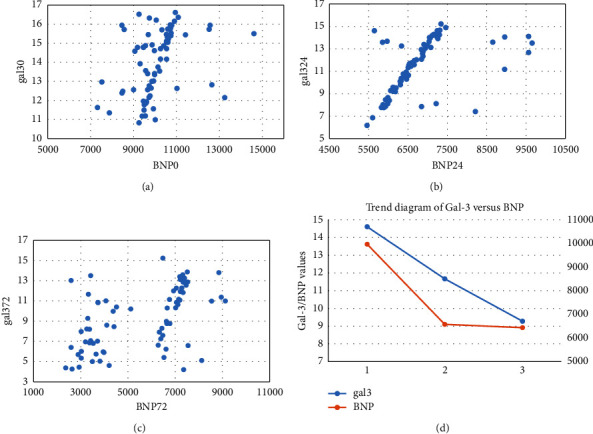
Four small plots to explore the relationship between galectin-3 (Gal-3) and B-type natriuretic peptide (BNP): (a) correlation analysis between Gal-3-T0 and BNP-T0; (b) correlation analysis between Gal-3-T24 and BNP-T24; (c) correlation analysis between Gal-3-T72 and BNP-T72; (d) Gal-3 and BNP mean trend chart.

Pearson correlation analysis was used to examine the relationship between Gal-3 and BNP at three time points in the 73 patients. There was a significant positive correlation between Gal-3 and BNP at each time point (*p* < 0.05), which is consistent with the average trend results of Gal-3 and BNP (as shown in Figure 2(d)). Specifically, Gal-3-T0 (baseline) was positively correlated with BNP-T0 (*r* = 0.373; *p* = 0.01) (as shown in Figure 2(a)), Gal-3-T24 (24 h) was positively correlated with BNP-T24 (*r* = 0.510; *p* < 0.01) (as shown in Figure 2(b)), and Gal-3-T72 (72 h) was positively correlated with BNP-T72 (*r* = 0.523; *p* < 0.01) (as shown in Figure 2(c)).

### 5.1. Analysis of the Risk Factors Affecting the Prognosis of ECMO Patients

Variables with *p* < 0.05 in the univariate analysis were included in a binary logistic regression analysis using the backward stepwise method, as shown in Table 3. The results showed that APACHE II score, Gal-3-T72, and BNP-T72 were independent risk factors for death in patients undergoing VA-ECMO for CS (*p* < 0.05), while platelets were not statistically significant (*p* > 0.05).

### 5.2. The Predictive Value of ROC Curve Analysis of Risk Factors on the Prognosis of VA-ECMO Patients

**Figure 3:**
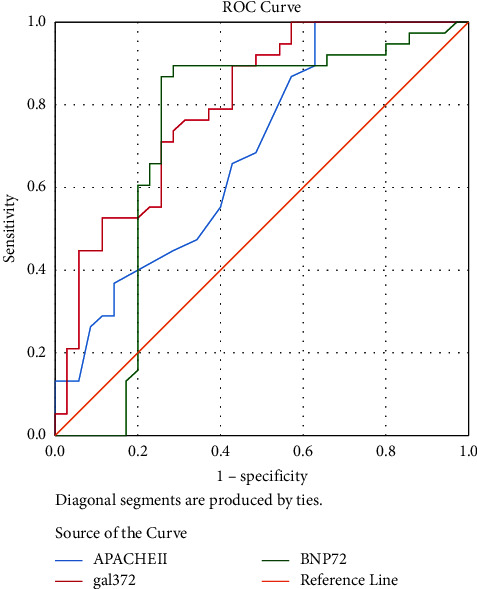
Receiver operating characteristic (ROC) curve of the predictive model and single index in predicting the prognosis of patients with venoarterial extracorporeal membrane oxygenation (VA-EMCO).

The independent risk factors, APACHE II score, Gal-3-T72, and BNP-T72, identified by the multivariate analysis in Table 3 are plotted as ROC curves, and the area under the curve (AUC) was calculated by software. The results are shown in Figure 3, where all three risk factors had significant predictive value for mortality (*p* < 0.05). The AUCs of the APACHE II score, Gal-3-T72, and BNP-T72 were 0.687, 0.799, and 0.723, respectively. The optimal cut-off point was determined by the maximum Youden index, which corresponded to the largest AUC for Gal-3-T72. The sensitivity and specificity of Gal-3-T72 > 7.165 ng/ml were 0.895 and 0.429, respectively (as shown in Table 4).

### 5.3. Construction of a Prediction Model for Death in Patients Undergoing VA-ECMO for CS and Evaluation of Its Value

**Figure 4:**
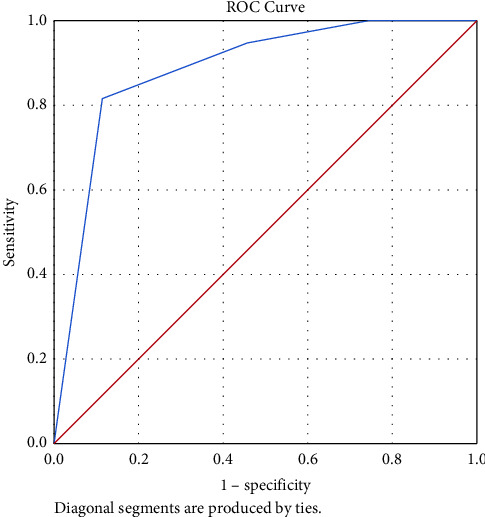
Receiver operating characteristic (ROC) curve of the death warning score in predicting the prognosis of patients with cardiogenic shock.

The APACHE II score, Gal-3-T72, and BNP-T72 were assigned based on the optimal threshold points, as detailed in Table 5. The death prediction score was constructed as APACHE II score + Gal-3-T72 + BNP-T72, with a total score ranging from 0 to 3. The ROC curve was plotted after assigning the raw data of the APACHE II score, Gal-3-T72, and BNP-T72 and calculating the death prediction score (as shown in Figure 4). The optimal threshold point was 2.5. A score >2.5 had 81.6% sensitivity and 70.2% specificity for predicting mortality (AUC: 0.884; 95% confidence interval (CI): 0.803–0.965; *p* < 0.05) with 81.6% positive predictive value, 88.5% negative predictive value, and 84.9% accuracy. Therefore, patients with CS undergoing VA-ECMO with a score >2 were classified as high-risk for the nonsurvivor group, while those with a score ≤2 were classified as low-risk for the nonsurvivor group.

## 6. Discussion

This study found that Gal-3 can be used to determine the prognosis of patients with CS undergoing VA-ECMO. The main findings of this study were as follows: (1) BNP and Gal-3 levels decreased significantly in patients with CS after VA-ECMO initiation, indicating that the ECMO technique is effective for treating patients with CS; (2) serum Gal-3 levels correlated well with serum BNP levels, suggesting that Gal-3 may be used to predict the degree of HF; (3) Gal-3 levels in the survivor and nonsurvivor groups were statistically different, indicating that Gal-3 can be used to predict the prognosis of patients with CS; and (4) the VA-ECMO mortality risk assessment model consisting of Gal-3-T72, BNP-T72, and the APACHE II score showed good predictive ability, with scores >2 belonging to the high-risk group for mortality and scores ≤2 to the low-risk group for mortality.

During VA-ECMO treatment, we observed that VA-ECMO provided arterial pressure support to the patient, which altered the original physiological circulatory structure and changed the preload and afterload of the heart [7]. This made it challenging to evaluate cardiac function using conventional examination methods. Qin et al. [8] studied the relationship between serum BNP and endothelin-1 (ET-1) levels and left atrial pump function in HF patients and found that BNP and ET-1 were negatively correlated with the left atrial ejection fraction, left atrial passive emptying fraction, and left atrial active emptying fraction (*p* < 0.05). Similarly, we also assessed cardiac function by BNP levels. Our study found that BNP levels at 24 h after ECMO treatment decreased significantly compared to before ECMO treatment, and the difference was statistically significant, indicating that VA-ECMO was beneficial for cardiac treatment in patients with CS [9]. ECMO can provide rapid circulatory support and protect organ perfusion, allowing time for cardiac recovery and preventing multiple organ failure [10]. However, the extent of BNP reduction in the late stage of ECMO depends on the patient's baseline cardiac function and recovery. In our sample, we observed that Gal-3 levels decreased significantly during ECMO treatment, and we hypothesized that the changes in Gal-3 might be related to the improvement of cardiac function in the patients. We further examined the relationship between BNP and Gal-3 using Pearson correlation analysis and found a strong correlation between BNP and Gal-3 at all three time points, with the strongest correlation between BNP and Gal-3 at 72 h (*r* = 0.523, *p* < 0.01), implying that serum Gal-3 could predict cardiac function. These results are consistent with Mahmoud U. Sani's observation [11]. Interestingly, despite peripheral arterial cannulation being the exclusive method for ECMO cannulation via the peripheral arteries, a study conducted by Fausto Biancari et al. examined the cannulation methods of 1269 patients requiring postcardiotomy VA-ECMO, which found that central arterial cannulation was associated with a higher risk of in-hospital mortality compared to peripheral arterial cannulation [12]. Our ECMO team will pay attention to and collect cases with both types of cannulation in the future.

We then analyzed the basic characteristics between the survivor and nonsurvivor groups, and the results showed intergroup differences in the APACHE II score, Gal-3, BNP, and platelets. In a study by Franziska Kaestner et al. [13], they analyzed 51 ECMO cases (including 38 VV-ECMO, 11 VAV-ECMO, and 2 VA-ECMO cases) and found a statistically significant difference in lactate levels between the survivor and nonsurvivor groups. The mean value for the survivor group was 2.0 (range: 0.4–13.5) and for the nonsurvivor group it was 4.0 (range: 1–21). However, our data showed no significant difference in lactate levels at 72 h between the two groups. We believe that the difference in results is related to the study subjects we included. Our 73 cases were all patients with cardiac failure without severe pulmonary failure symptoms, and VA-ECMO can quickly relieve patients' ischemic-hypoxic symptoms with a significant treatment effect. On the other hand, the main study subjects included by Franziska Kaestner's team were administered VV-ECMO treatment for pulmonary failure. VV-ECMO poorly relieves ischemic-hypoxic symptoms in such patients compared with patients with CS, which may have led to inconsistent results.

In the multivariate model, we selected Gal-3-T72 and BNP-T72 as risk factors for analysis. The results showed that the APACHE II score, Gal-3-T72, and BNP-T72 were independent risk factors for death in patients undergoing VA-ECMO for CS. The ROC analysis showed that all three factors had significant predictive power for VA-ECMO patients, and the AUC of Gal-3-T72 was the largest (0.799). Patients with CS are characterized by organ ischemia, hypoxia, inflammatory factor disorders, hemodynamic changes, and volume and pressure changes in the heart [14–17]. Cardiac failure during the state of CS promotes the release of serum inflammatory factors, macrophage activation, and migration to cardiac tissue, leading to a significant increase in Gal-3 that promotes cardiomyocyte fibrosis [18, 19]. When patients are given VA-ECMO support therapy, ECMO shares the workload done by the heart, resulting in the alleviation of cardiac failure symptoms, a decrease in the release of inflammatory factors, and a gradual decrease in Gal-3 levels.

There are limited models for predicting ECMO outcomes, and the predictive variables differ for different populations. Because more than half of the patients in our ECMO cohort had cardiovascular disease, we sought to establish a prediction model for patients with CS. AUC of the model comprising the APACHE II score, Gal-3-T72, and BNP-T72 was 0.884, and the model showed higher predictive accuracy than each risk factor alone. Patients with CS on VA-ECMO who scored >2 were categorized as high-risk for mortality, and those who scored ≤2 were categorized as low-risk for mortality. This study indicates that the combination of the APACHE II score, Gal-3-T72, and BNP-T72 can be applied to predict the outcomes of VA-ECMO and is feasible. In contrast, Franziska Kaestner included too many types of ECMO in the construction of their ECMO prediction model, including VV-ECMO, VAV-ECMO, and VA-ECMO [13]; thus, their model is not well suited for patients with CS.

Schmidt et al. developed the SAVE score to assess the prognosis of refractory CS, based on factors including the cause of CS, age, organ function, and blood pressure. They found that the SAVE score performed better than several standard ICU severity scores [20]. Mohamed Laimoud et al. found that increasing the ∆1 SOFA score (odds ratio (OR) = 2.506, 95% CI: 1.681–3.735, *p* < 0.001) and increasing the blood lactate level (OR = 1.388, 95% CI: 1.015–1.898, *p* = 0.04) were significantly associated with hospital mortality after VA-ECMO support for adults with CS [21].

The scoring system we used in our study is relatively simple and includes a limited number of indicators. However, it has proven to be more valuable in predicting mortality in ECMO patients than single indicators. Therefore, this scoring system may have clinical significance for patient evaluation. In future research, we hope to further investigate this by incorporating measures such as the SAVE score, SOFA score, and peak lactate levels.

There are several limitations to this study. First, our team failed to register the clinical trial, potentially leading to biased patient selection and jeopardizing the accuracy and applicability of our research outcomes. We will further improve our research process to ensure that our future studies comply with relevant regulations and ethical requirements. Second, the small sample size may cause possible bias in the statistical analysis and requires further multicenter and large sample studies to validate the results. Third, most of the models constructed by our team are based on patients with cardiovascular disease and may not be suitable for use in VV-ECMO or VAV-ECMO patients. Further studies are needed to investigate the applicability of our model in these patient populations. Unfortunately, peak lactate levels were not included as an analytical measure. Peak lactate levels are a more sensitive indicator for assessing tissue perfusion and can more accurately predict mortality in patients with VA-ECMO. Mohamed Laimoud and Mosleh Alanazi's study also supports this viewpoint; their research has shown that peak lactate levels had better performance (AUC = 0.889; 95% CI: 0.825–0.953; *p* < 0.001), and that lactate levels after 24 h of ECMO initiation performed best in terms of sensitivity and specificity for discriminating mortality (AUC 0.93; 95% CI: 0.878–0.983; *p* < 0.001) [10].

## 7. Conclusion

Gal-3 may be used as a predictor of prognosis in patients on VA-ECMO for CS. The VA-ECMO early warning score, which includes the APACHE II score, Gal-3-T72, and BNP-T72, has greater predictive value for mortality in ECMO patients than single indicators. This scoring system has the potential to provide valuable prognostic information for VA-ECMO patients. However, further validation of its generalizability is required with a larger sample size and broader population.

## Figures and Tables

**Table 1:** Baseline characteristics of studied patients with venoarterial extracorporeal membrane oxygenation (VA-EMCO).

	All	Survivors (*n* = 35, 47.95%)	Nonsurvivors (*n* = 38, 52.05%)	*z*/*χ*/*t*	*p*
Gender				0.47	0.829
Male	53 (72.61)	25 (34.25)	28 (38.36)		
Female	20 (27.4)	10 (13.7)	10 (13.7)		
Age	57 (48–67)	56 (47,67)	60 (51.25, 66.25)	−0.354	0.724
APACHE II	27.63 (±6.65)	24.94 ± 1.226	30.11 ± 0.802	−3.523	0.001
BMI >35 kg/m^2^ (%)	29 (39.73)	13 (17.81)	16 (21.92)	0.187	0.665
Assist IABP (%)	27 (36.99)	15 (20.55)	12 (16.44)	0.994	0.319
Disease (%)					
Acute myocardial infarction	24 (32.88)	14 (19.18)	10 (13.7)	11.923	0.012
Sudden cardiac death	2 (2.74)	2 (2.74)	0	14.362	0.003
Aortic dissection (type A)	16 (21.92)	4 (5.48)	12 (16.44)	14.904	0.002
Valvular heart disease	21 (28.77)	11 (15.07)	10 (13.7)	10.721	0.025
Infectious endocarditis	3(4.11)	3 (4.11)	0	15.112	0.001
Dilated cardiomyopathy	2 (2.74)	0	2 (2.74)	14.029	0.007
Exotic myocarditis	1 (1.37)	0	1 (1.37)	14.056	0.027
Other	4 (5.48)	1 (1.37)	3 (4.11)	12.672	0.021
Basic disease (%)					
Type 2 diabetes	15 (20.55)	6 (8.22)	9 (12.33)	11.174	0.022
Hypertension	31 (42.47)	10 (13.7)	21 (28.77)	15.547	0.001
Hyperlipemia	11 (15.07)	6 (8.22)	5 (6.85)	11.202	0.022
AKI	62 (84.94)	26 (35.62)	36 (49.32)	5.954	0.015
Hemodialysis	50 (68.5)	18 (24.66)	32 (43.84)	9.073	0.03
Perfusion tube distal to the limb	67 (91.79)	31 (42.47)	36 (49.32)	0.918	0.338
Lower limb ischemia	9 (12.33)	2 (2.74)	7 (9.59)	2.722	0.099
Gastrointestinal bleeding	26 (35.62)	11 (15.07)	15 (20.55)	0.514	0.473
LVEF%	30.14	31.91	28.5	2.411	0.019
Gal-3-T0 (ng/ml)	14.61 (12.56–15.48)	14.16 (12.48, 15.44)	14.955 (12.6275, 15.725)	−1.336	0.181
Gal-3-T24	11.67 (9.395, 13.595)	10.1 (8.12, 11.75)	13.095 (11.32, 14.02)	−4.135	0.001
Gal-3-T72	9.28 (6.61, 11.875)	6.85 (5.34, 10.82)	11.105 (8.645, 12.87)	−4.395	0.001
BNP-T0 (pg/ml)	9970 (9494–10596)	9738 (9305, 10530)	10197.5 (9639.25, 10643.25)	−1.623	0.105
BNP-T24	6574 (6134.5, 7096)	6300 (5928, 6660)	6895 (6493.25, 7165.25)	−3.285	0.001
BNP-T72	6437 (3511.5, 7218)	3725 (3296, 6524)	6976.5 (6374, 7289.5)	−3.28	0.001
Plt	85 (57.50–117)	93 (72, 127)	66.5 (43.5, 108.25)	−2.612	0.009
Lac		2.1 (1.4, 3.4)	2.45 (1, 3.925)	−0.464	0.643
T_ECMO_	5.8 (4.62–7.57)	5.96 (4.54, 7.73)	5.69 (4.745, 7.365)	−0.353	0.724
T_ICU_	19.04 (13.92–26.15)	19.39 (15.31, 24.16)	18.78 (13.8225, 26.57)	−0.166	0.868

Normal distribution of measurement data is expressed as mean ± standard deviation (mean ± SD). Non-normal distribution is represented by the median, interquartile spacing (M (QL, QU)). Gal-3-T0 and BNP-T0 showed blood samples before ECMO, Gal-3-T24 and BNP-T24 at 24 h after ECMO, and Gal-3-T72 and BNP-T72 at 72 h after ECMO. T_ECMO_ refers to the duration of ECMO treatment patients, and T_ICU_ refers to the duration of the ICU stay.

**Table 2:** Galectin-3 (Gal-3) and B-type natriuretic peptide (BNP) Friedman analysis.

Sample samples	Test statistic	*p*
Gal-3-T0, Gal-3-T24	0.92	<0.01
Gal-3-T0, Gal-3-T72	1.92	<0.01
Gal-3-T24, Gal-3-T72	1	<0.01
BNP-T0, BNP-T24	1.48	<0.01
BNP-T0, BNP-T72	1.52	<0.01
BNP-T24, BNP-T72	0.04	0.81

**Table 3:** Binary logistic analysis of risk factors for patients with extracorporeal membrane oxygenation.

	*p*	OR	95% CI	
APACHE II	0.022	1.153	1.02	1.302
Gal-3-T72	0.045	1.279	1.005	1.627
BNP-T72	0.049	1.001	1	1.001
Plt	0.097	0.986	0.97	1.003
Constant	0.004	0.001		

**Table 4:** Receiver operating characteristic (ROC) curves analyzed the predictive value of APACHE II, galectin-3 (Gal-3), and B-type natriuretic peptide (BNP).

	AUC	Cut-off values	Sensitivity	Specificity	Youden index
APACHE II	0.687	21.5	1	0.629	0.371
Gal-3	0.799	7.165	0.895	0.429	0.466
BNP	0.723	4461.5	0.868	0.257	0.611

**Table 5:** Death early warning rating table.

	Score
APACHE II ≤ 21.5	0
APACHE II > 21.5	1
Gal-3-T72 ≤ 7.165	0
Gal-3-T72 > 7.165	1
BNP-T72 ≤ 4461.5	0
BNP-T72 > 4461.5	1

## Data Availability

The data used to support the findings of this study are included within the article (see the link of the public database https://doi.org/10.6084/m9.Figshare.21432435.v2 for details).

## Abbreviations

Gal-3: Galectin-3
CS: Cardiogenic shock
AMI: Acute myocardial infarction
ECMO: Extracorporeal membrane oxygenation
VA-ECMO: Venoarterial-extracorporeal membrane oxygenation
APTT: Activated partial thromboplastin time
AUC: Area under curve.

## References

[1] Reynolds H. R., Hochman J. S. (2008). Cardiogenic shock: current concepts and improving outcomes. *Circulation*.

[2] Berg D. D., Bohula E. A., Morrow D. A. (2021). Epidemiology and causes of cardiogenic shock. *Current Opinion in Critical Care*.

[3] Rathod K. S., Koganti S., Iqbal M. B. (2018). Contemporary trends in cardiogenic shock: incidence, intra-aortic balloon pump utilisation and outcomes from the London Heart Attack Group. *European Heart Journal: Acute Cardiovascular Care*.

[4] Thiele H., Allam B., Chatellier G., Schuler G., Lafont A. (2010). Shock in acute myocardial infarction: the Cape Horn for trials?. *European Heart Journal*.

[5] Va A., Zaslavskaya E. L., Soboleva A. V. (2016). Galectin-3 in patients with paroxysmal and persistent atrial fibrillation and metabolic syndrome. *Kardiologiia*.

[6] Sharma U. C., Pokharel S., Van Brakel T. J. (2004). Galectin-3 marks activated macrophages in failure-prone hypertrophied hearts and contributes to cardiac dysfunction. *Circulation*.

[7] Kulkarni T., Sharma N. S., Diaz-Guzman E. (2016). Extracorporeal membrane oxygenation in adults: a practical guide for internists. *Cleveland Clinic Journal of Medicine*.

[8] Qin L., Liu X., Li Y. (2020). Correlation of serum BNP and ET-1 levels with cardiac pump function and ventricular remodeling in patients with heart failure. *Cellular & Molecular Biology*.

[9] Huang S., Wu E., Ko W. (2006). Clinical implication of blood levels of B-type natriuretic peptide in pediatric patients on mechanical circulatory support. *The Annals of Thoracic Surgery*.

[10] Laimoud M., Alanazi M. (2020). The clinical significance of blood lactate levels in evaluation of adult patients with veno-arterial extracorporeal membrane oxygenation. *The Egyptian Heart Journal*.

[11] Sani M. U., Damasceno A., Davison B. A. (2021). N‐terminal pro BNP and galectin‐3 are prognostic biomarkers of acute heart failure in sub‐Saharan Africa: lessons from the BAHEF trial. *ESC Heart Failure*.

[12] Biancari F., Kaserer A., Perrotti A. (2022). Central versus peripheral postcardiotomy veno-arterial extracorporeal membrane oxygenation: systematic review and individual patient data meta-analysis. *Journal of Clinical Medicine*.

[13] Kaestner F., Rapp D., Trudzinski F. C. (2018). High serum bilirubin levels, NT-pro-BNP, and lactate predict mortality in long-term, severely ill respiratory ECMO patients. *ASAIO Journal*.

[14] Tehrani B. N., Truesdell A. G., Psotka M. A. (2020). A standardized and comprehensive approach to the management of cardiogenic shock. *JACC Heart Failure*.

[15] Harjola V. P., Lassus J., Sionis A. (2015). Clinical picture and risk prediction of short‐term mortality in cardiogenic shock. *European Journal of Heart Failure*.

[16] Jentzer J. C., Hernandez-Montfort J. (2023). Refining the stratification and prognosis of cardiogenic shock patients to improve their outcome. *Canadian Journal of Cardiology*.

[17] Sperry A. E., Williams M., Atluri P. (2021). The Surgeon’s role in cardiogenic shock. *Current Heart Failure Reports*.

[18] Ibarrola J., Arrieta V., Sádaba R. (2018). Galectin-3 down-regulates antioxidant peroxiredoxin-4 in human cardiac fibroblasts: a new pathway to induce cardiac damage. *Clinical Science*.

[19] Dumic J., Dabelic S., Flögel M. (2006). Galectin-3: an open-ended story. *Biochimica et Biophysica Acta (BBA)- General Subjects*.

[20] Schmidt M., Burrell A., Roberts L. (2015). Predicting survival after ECMO for refractory cardiogenic shock: the survival after veno-arterial-ECMO (SAVE)-score. *European Heart Journal*.

[21] Laimoud M., Alanazi M. (2020). The validity of SOFA score to predict mortality in adult patients with cardiogenic shock on venoarterial extracorporeal membrane oxygenation. *Critical Care Research and Practice*.

